# Targeted encouragement of GP consultations for possible cancer symptoms: a randomised controlled trial

**DOI:** 10.3399/bjgp20X713489

**Published:** 2020-04-20

**Authors:** Jean-Pierre Laake, Daniel Vulkan, Samantha L Quaife, William T Hamilton, Tanimola Martins, Jo Waller, Dharmishta Parmar, Peter Sasieni, Stephen W Duffy

**Affiliations:** Centre for Cancer Prevention, Wolfson Institute of Preventive Medicine, Queen Mary University of London, London; medical student, Warwick Medical School, University of Warwick, Coventry.; Centre for Cancer Prevention, Wolfson Institute of Preventive Medicine, Queen Mary University of London, London.; Centre for Cancer Prevention, Wolfson Institute of Preventive Medicine, Queen Mary University of London, London; senior research fellow, Research Department of Behavioural Science and Health, Institute of Epidemiology and Health Care, University College London, London.; College of Medicine and Health, University of Exeter, Exeter.; College of Medicine and Health, University of Exeter, Exeter.; Research Department of Behavioural Science and Health, Institute of Epidemiology and Health Care, University College London, London; reader in cancer behavioural science, School of Cancer & Pharmaceutical Sciences, King’s College London, London.; Centre for Cancer Prevention, Wolfson Institute of Preventive Medicine, Queen Mary University of London, London.; King’s College London, London.; Centre for Cancer Prevention, Wolfson Institute of Preventive Medicine, Queen Mary University of London, London.

**Keywords:** cancer, diagnosis, general practice, health promotion, primary health care, randomized controlled trial

## Abstract

**Background:**

For some common cancers, survival is lower in the UK than in comparable high-income countries.

**Aim:**

To assess the effectiveness of a targeted postal intervention (to promote awareness of cancer symptoms and earlier help seeking) on patient consultation rates.

**Design and setting:**

A two-arm randomised controlled trial was carried out on patients aged 50–84 years registered at 23 general practices in rural and urban areas of Greater London, Greater Manchester, and the North East of England.

**Method:**

Patients who had not had a consultation at their general practice in the previous 12 months and had at least two other risk factors for late presentation with cancer were randomised to intervention and control arms. The intervention consisted of a posted letter and leaflet. Primary outcome was the number of consultations at the practice with patients randomised to each arm in the 6 months subsequent to posting the intervention. All patients with outcome data were included in the intention-to-treat analyses.

**Results:**

In total, 1513 patients were individually randomised to the intervention (*n* = 783) and control (*n* = 730) arms between Nov 2016 — May 2017; outcome data were available for 749 and 705 patients, respectively, with a statistically significantly higher rate of consultation in the intervention arm compared with the control arm: 436 versus 335 consultations (relative risk 1.40, 95% confidence interval = 1.11 to 1.77, *P* = 0.004). There was, however, no difference in the numbers of patients consulting.

**Conclusion:**

Targeted interventions of this nature can change behaviour; there is a need to develop interventions that can be more effective at engaging patients with primary care. This study demonstrates that targeted interventions promoting both awareness of possible cancer symptoms and earlier health seeking, can change behaviour. There is a need to develop and test interventions that can be more effective at engaging the most at-risk patients.

## INTRODUCTION

For some common cancers (including lung, colorectal, and breast cancer), survival in the UK is lower than in comparable high-income countries.^1^ It is thought that this is due largely to later-stage disease at diagnosis in the UK. This may be partly related to system delays following presentation, some of which have increased over the last decade in England;^2^ it may also arise from low symptom awareness, negative beliefs about cancer, and reluctance to ‘waste the doctor’s time’^3^^,^^4^ — all of which could extend the patient interval.^5^ There is a positive association between levels of awareness of cancer and survival.^6^ In addition, there remain geographic and socioeconomic inequalities in cancer survival, although the gap has narrowed somewhat in recent years.^7^^,^^8^

Several campaigns, including Be Clear on Cancer, have aimed to raise the level of symptom awareness and encourage help seeking for symptoms.^9^ Some have shown encouraging results with respect to diagnosis of cancer at an earlier stage;^10^^,^^11^ others have shown an increase in consultations and referrals, but no effect on cancer diagnoses or the stage of disease at diagnosis.^12^ This gives rise to the concern that population-level, mass-media campaigns may increase consultations among the ‘worried well’, rather than reaching the population most in need of earlier presentation.

This concern led to speculation that targeted, rather than whole-population, symptom-awareness interventions might be more effective at reducing the patient interval and improving the disease stage at symptomatic presentation. Targeting, in this context, is at those whose circumstances or lifestyle imply that, should they develop cancer, they will be at increased risk of being diagnosed at a later stage in the disease trajectory. This population includes: those of lower socioeconomic status, smokers, those with chronic comorbidities, certain ethnic groups, and those who tend to use emergency services rather than primary care.^13^^–^17

**Table:** How this fits in

Later stage of cancer diagnosis is associated with poorer survival, and may arise from low symptom awareness and delays in presenting to primary care. Population-wide campaigns to increase awareness and encourage help seeking have shown mixed results in terms of stage at diagnosis and numbers of primary care consultations. This randomised controlled trial was targeted at a population whose circumstances suggested that, should they develop cancer, they would be at increased risk of being diagnosed with later-stage disease. This study demonstrates that targeted interventions of this nature, promoting both awareness of cancer symptoms and earlier health seeking, can change consultation behaviour of those who are likely to benefit most from earlier symptomatic presentation. However, in his study the intervention increased the frequency of consultation but not the number of persons consulting.

## METHOD

### Study design and setting

A two-arm randomised controlled trial (RCT) — called Writing to Encourage Late Consultation Outpatients to Make Engagement with their GP (WELCOME-GP) — was carried out to test the effectiveness of a postal intervention that was targeted at certain vulnerable groups and intended to promote awareness of cancer symptoms and earlier help seeking at general practice. It was designed to:

- promote awareness of six cancer ‘red-flag’ symptoms; and
- allay fears of wasting the doctor’s time.

The intervention was targeted at a population that had not attended their general practice in the previous 12 months, with factors that suggest this may be incongruous with their clinical need and attributes known to be risk factors for late-stage presentation of cancer.^13^^–^17 A total of 24 practices from three areas in England — rural and urban areas of the North East, Greater Manchester, and Greater London — agreed to take part; one practice subsequently withdrew.

### Sample and randomisation

Patients were eligible for inclusion if they:

- were aged 50–84 years;
- were registered with a participating practice;
- had not had a consultation at their registered practice in the previous 12 months; and satisfied at least two of the following:
  — lower socioeconomic status (in either of the two lowest quintiles of the Index of Multiple Deprivation 2015, based on post code);^18^
  — missed last scheduled screening for breast cancer, bowel cancer, cervical cancer, or abdominal aortic aneurism;
  — history of use of emergency or out-of-hours (OOH) services instead of primary care;
  — missed last appointment for chronic-disease monitoring/management;
  — lived alone (indexed by being the only person registered with the practice at their address) as a marker of social isolation; and
  — smoker (ever).

Eligibility was assessed from patients’ primary care electronic health records. It was not possible to identify, with confidence, patients who had used emergency or OOH services from the practice databases so no patients were recruited on this basis.

Persons were ineligible if:

- they already had a diagnosis of cancer;
- the GP considered recruitment inappropriate in view of the patient’s state of health or cognitive capacity; or
- the GP felt that the patient would not wish to be included in the research.

Sample-size calculations are described in Supplementary Appendix S1. Each practice was provided with a computer-generated randomisation schedule. Practice staff identified eligible patients and randomly allocated them to the intervention or to usual care; there was no concealment of allocation. At the end of the 6-month observation period, staff at each practice reported the following for each randomised patient:

- the number of consultations by each patient who continued to be registered throughout the period;
- the month of the consultation(s);
- the reason for the consultation(s);
- whether any investigations or physical examinations were arranged and completed; and
- whether referrals to secondary care were made.

De-identified data were stored on encrypted databases that remained at practices. Intention-to-treat (ITT) analyses were carried out by the data analysis team on anonymised data for all eligible patients for whom data were available at the end of the observation period. Due to the nature of the intervention, it was necessary that patients in both groups were unaware that they were taking part in a research study; however, it would have been impractical for practice staff and the data analysis team to operate on this basis so they were not blinded to the allocation. Censored, anonymised data output was generated from the encrypted database at each practice and securely transmitted to the research team.

### Intervention

The Model of Pathways to Treatment provides a framework for understanding the pathways between an individual first detecting a bodily change and subsequently undergoing treatment,^19^ beginning with ‘appraisal’ and ‘help seeking’ intervals. Crucially, the individual must believe there to be a reason to present with their symptom; this is often motivated by heuristics, such as concern about a worsening symptom or its interference with their daily activities.^20^

The present intervention can be contextualised within these intervals, in that it was intended to:

- prompt appraisal through symptom awareness;
- provide individuals with a ‘cue to action’ for visiting their GP; and
- use messaging to counteract known attitudinal barriers to help seeking.

Furthermore, the personalised, GP-led approach drew on the success of primary care endorsement in the cancer-screening context.^21^^,^^22^

The intervention consisted of a letter (see Supplementary Appendix S2), signed by one of the GPs at the patient’s practice, noting that the recipient had not been seen at the practice for some time and reassuring them that consulting with any symptoms would not be considered wasting the doctor’s time. The letter also drew attention to an enclosed leaflet (see Supplementary Appendix S3), which detailed six symptoms that are considered to potentially raise suspicion for cancer:

- blood in urine;
- blood in stool;
- persistent cough;
- haemoptysis;
- difficulty swallowing; and
- unexplained weight loss.

These symptoms were chosen as they feature in the National Institute for Health and Care Excellence’s recommendations for referral for suspected cancer.^23^ The first practice mailed the intervention materials to all patients allocated to the intervention arm on 11 November 2016, and the last did so on 31 May 2017.

### Outcomes

The primary endpoint was the equivalent annual rate of consultations with clinicians from the patient’s registered practice in the 6 months following randomisation (that is, the date that letters were mailed), whether the consultations were at the practice itself, home visits, or telephone consultations. This rate was calculated as the total number of consultations divided by the total period of exposure (half a year per patient; only those patients registered at the practice for the full 6 months were included in the analysis). Secondary endpoints were:

- total general practice activity — that is, consultations, referrals to secondary care, completed investigations (for example, blood tests) arranged by the practice, physical examinations carried out at the practice (for example, blood pressure check and digital rectal examination), and any diagnoses; and
- the use of emergency and OOH services in the study and control groups.

As noted above, it could not be established with confidence whether patients had made use of emergency services, so this particular analysis was not possible. It also became clear during the course of the study that it would not be possible to collect data on subsequent diagnoses, as these may have occurred some time after the end of the 6-month follow-up. Several other analyses were carried out: the primary and secondary analyses were repeated, considering only those consultations that had taken place for at least one of the six symptoms identified in the leaflet; the total number of consultations that took place in the same calendar month as randomisation (the period immediately following the mailing of the intervention) were compared between the intervention and control groups.

### Statistical analyses

All randomised patients for whom follow-up data were available were included in the ITT analyses. Data were analysed by zero-inflated Poisson regression,^24^ a technique that takes account of the large number of persons with no consultations at all during the 6-month observation period. Relative risks (RRs) of consultation with 95% confidence intervals (CIs) were calculated. Robust variance estimators were used, allowing for clustering by person. Stata (version 15.1) was used for data analysis.

## RESULTS

**Figure 1.**
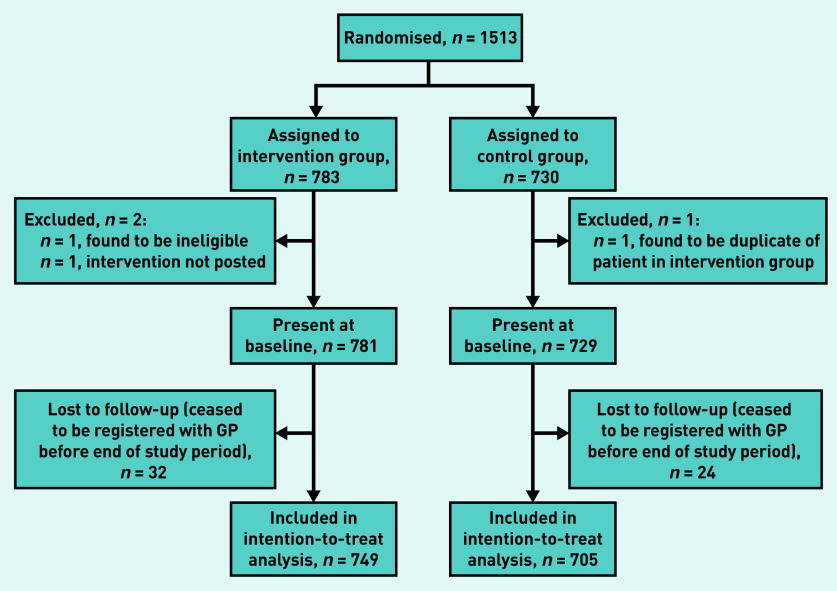
*WELCOME-GP trial: CONSORT flow diagram.*

A total of 1513 patients were randomised using simple 1:1 randomisation (Figure 1). Table 1 shows the age, sex, eligibility criteria satisfied, and region of residence of the patients present at baseline; there were no differences between the intervention and control arms at a nominal 5% level of significance. Of the 1513 randomised, 1454 (96.1%) (intervention: *n* = 749, control: *n* = 705) remained at the same practice 6 months post-randomisation and, therefore, had a primary endpoint for analysis.

**Table 1.** Baseline characteristics of study sample, *N* = 1510

**Factor**	**Intervention, *n* (%)**	**Control, *n* (%)**
Total sample	781	729

**Age, years**		
<50	0 (0.0)	0 (0.0)
50–59	621 (79.5)	557 (76.4)
60–69	104 (13.3)	113 (15.5)
≥70	56 (7.2)	59 (8.1)

**Sex**		
Male	546 (69.9)	500 (68.6)
Female	235 (30.1)	229 (31.4)

**Lower socioeconomic status**		
No	115 (14.7)	118 (16.2)
Yes	666 (85.3)	611 (83.8)

**Missed last screening appointment**		
No / not applicable	565 (72.3)	522 (71.6)
Yes	216 (27.7)	207 (28.4)

**Missed last chronic disease monitoring appointment**		
No	628 (80.4)	610 (83.7)
Yes	153 (19.6)	119 (16.3)

**Living alone**		
No	394 (50.4)	381 (52.3)
Yes	387 (49.6)	348 (47.7)

**Smoker**		
No	84 (10.8)	81 (11.1)
Yes	697 (89.2)	648 (88.9)

**Region**		
North East England	241 (30.9)	221 (30.3)
Greater Manchester	280 (35.9)	273 (37.4)
Greater London	260 (33.3)	235 (32.2)

### Statistics

#### Primary analyses

Table 2 shows general practice consultations and onward referrals in the two trial arms, and the reasons given for consultations. There was a significantly higher rate of consultation in the intervention arm compared with the control arm (*n* = 436 consultations versus *n* = 335 consultations, RR 1.40, 95% CI = 1.11 to 1.77, *P* = 0.004), but no statistically significant difference between arms in the likelihood of individuals consulting (odds ratio 0.92, 95% CI = 0.72 to 1.18, *P* = 0.53). Onward referral rates were higher in the intervention arm, but there was no statistical significance (*n* = 85 [19.5% of consultations] versus *n* = 56 [16.7%], RR 1.44, 95% CI = 0.97 to 2.14, *P* = 0.070). There was a statistically significant difference in the number of clinical investigations and physical examinations carried out as a result of consultations, with 282 in the intervention arm compared with 212 in the control arm (RR 1.34, 95% CI = 1.01 to 1.77, *P* = 0.041).

**Table 2.** Consultations, reasons for consultation, onward referrals to secondary care, and completed clinical investigations and examinations in the intervention and control groups

**Consultation measure**	**Intervention, *n* (%)^a^**	**Control, *n* (%)^a^**	**Statistic (95% CI)^a^**	***P*-value**
Persons still registered at end of study	749	705	—	—

Persons consulting	165 (22.0)	165 (23.4)	OR^b^ 0.92 (0.72 to 1.18)	0.53

Total consultations	436 (1.16/person year)	335 (0.95/person year)	**RR^c^ 1.40 (1.11 to 1.77)**	**0.004**

**Reason for consultation**				
Blood in urine	1 (0.2)	0 (0.00)	N/A	—
Blood in stool	2 (0.5)	4 (1.2)	0.38 (0.07 to 2.13)	0.27
Persistent cough	30 (6.9)	15 (4.5)	1.54 (0.62 to 3.82)	0.36
Haemoptysis	0 (0.00)	0 (0.00)	N/A	—
Difficulty swallowing	2 (0.5)	2 (0.6)	0.77 (0.05 to 12.14)	0.85
Weight loss (unexplained)	3 (0.7)	1 (0.3)	2.31 (0.25 to 21.71)	0.47
Other	398 (91.3)	313 (93.4)	0.97 (0.92 to 1.03)	0.42

Secondary care referral	85 (19.5)	56 (16.7)	1.44 (0.97 to 2.14)	0.070

Clinical investigations and examinations	282 (64.7)	212 (63.3)	**1.34 (1.01 to 1.77)**	**0.041**

a *Unless otherwise specified.*

b *Logistic regression.*

c *Zero-inflated Poisson regression. Bold text denotes significance at an alpha level of 0.05. NA = not applicable due to insufficient data. OR = odds ratio. RR = relative risk.*

#### Additional analyses

The number of consultations for the symptoms described in the leaflet was higher in the intervention arm (*n* = 38) than in the control arm (*n* = 22) (Table 2), although this was not statistically significant (RR 1.74, 95% CI = 0.81 to 3.74, *P* = 0.16). Onward referral in relation to symptoms in the leaflet did not differ by arm (*n* = 8 versus *n* = 8, RR 0.88, 95% CI = 0.25 to 3.07, *P* = 0.84). There were more subsequent investigations and examinations in the intervention arm for symptoms in the leaflet (*n* = 34 versus *n* = 19) but this difference was also not statistically significant (RR 1.94, 95% CI = 0.90 to 4.21, *P* = 0.093). There were no statistically significant differences between the intervention and control arms with regard to the reasons given for consultation.

Table 3 shows the number of consultations in the calendar month of randomisation and in subsequent months. Table 4 shows the month in which the first consultation took place. There were statistically significantly more consultations during the calendar month of randomisation in the intervention arm (*n* = 16), than in the control arm (*n* = 8) (Table 4) (RR 2.50, 95% CI = 1.07 to 5.86, *P* = 0.035). However, there was no statistically significant difference between the arms, in terms of the month of first consultation (0.3 months earlier in intervention arm, 95% CI = −0.7 to 0.1, *P* = 0.10). Although the date of randomisation in each practice was known, exact dates of consultations were not. Sensitivity analyses determined that the date of the month on which randomisation occurred did not have any impact on the statistical significance of these results.

**Table 3.** Number of consultations in each calendar month, post-randomisation, in the intervention and control groups

**Calendar months since randomisation**	**Intervention, *n***	**Control, *n***
0^a^	20	8
1	74	51
2	43	51
3	84	73
4	80	66
5	70	54
6	65	32
Total number of consultations	436	335

a *Partial month, as randomisation took place on different days of the month in each practice.*

**Table 4.** Calendar month since randomisation in which first consultations took place, in the intervention and control groups.

**Calendar months since randomisation**	**Intervention, *n***	**Control, *n***
0^a^	16	8
1	37	34
2	20	25
3	40	31
4	23	28
5	15	24
6	14	15
No consultations within 6 months	584	540
Patients for whom data was available	749	705

a *Partial month, as randomisation took place on different days of the month in each practice.*

## DISCUSSION

### Summary

This trial took place in a population that was interacting less with primary care than most of their age-matched peers and had factors associated with a lesser tendency to seek help and associated with late presentation of cancer.^13^^–^17^,^^25^^,^^26^ The results demonstrated that a targeted primary care-based intervention can change consulting behaviour in this population, but not necessarily in the way expected: in the intervention group, there were statistically significantly more general practice consultations than in the control group during the 6-month intervention period but there was no increase in the number of *persons* consulting. More investigations and examinations were carried out in the intervention group and this difference was statistically significant. There were also more referrals in the intervention group compared to the control group, although this difference was not statistically significant. Given the relatively small proportion of the population targeted in this trial it is possible that, were more primary care providers incentivised to deliver such a targeted intervention, this might result in statistically and clinically significant numbers of increased primary care consultations for cancer ‘red-flag’ symptoms and referrals.

Irrespective of the symptom(s) with which patients presented, if the increase in consultation rates observed among the target patient group in this trial were to be sustained, this may result in earlier diagnoses of cancer; this is because GPs generally enquire about ‘red-flag’ symptoms when patients present with other lower-risk possible cancer symptoms. Increased consultation rates also provide GPs with an opportunity to: promote primary and secondary prevention of cancer and other disease by supporting patients to attend non-symptomatic screening;^27^ to manage other chronic conditions more effectively; and to adopt healthier behaviours through initiatives such as Making Every Contact Count.^28^ These are activities that GPs should already routinely perform for patients who present frequently,^29^ which may partially explain inequalities in outcomes in those groups that access services less often.

### Strengths and limitations

There were several strengths to this trial. The authors were able to recruit a relatively large sample of patients from typically hard-to-reach groups registered at both inner-city and rural practices across England, which gives confidence that the statistically significant difference in the number of consultations can be applied to the national population. This intervention was also relatively cheap and acceptable to most practices approached. Finally, the unusual design of the study — with no study-specific prior informed-consent process — meant the trial was not affected by selection bias for patients who were most interested in and prepared to alter behaviour.

Although the study was sufficiently powered to detect a difference in the number of consultations and persons consulting, it was not powered to be able to detect clinically significant differences in additional outcomes such as the number of consultations for specific symptoms in the leaflet. It was possible for the authors to measure attendance at general practice, investigations, examinations, and referrals to secondary care, but it was not possible to determine whether the results pertaining to those were due to the intended direct effects of the intervention on presenting behaviour, or whether clinicians were also affected by the intervention.

In addition, the authors were unable to assess the duration of effect and the impact on clinical end-points as a considerably larger study population would have been required to achieve sufficient power. Due to information-governance considerations, the authors were not able to collect outcome data that were linked to demographic data for stratified or adjusted analyses, which would have been underpowered. Such data should be collected in future studies assessing effects and impact on clinical endpoints should these have sufficient statistical power for such analyses.

Another limitation was the concept of changing the behaviour of a particularly difficult-to-reach group with a single postal communication; this proved to be rather ambitious. It is not known whether a second letter would increase effect or if there is a ‘dose response’ to this intervention. There are several stages in the process of cancer awareness, willingness to seek help, referral, and diagnosis; the authors considered they were not at the stage of sufficient knowledge to assess the impact of a psychosocial intervention on cancer diagnoses. Furthermore, a major limitation of targeted postal awareness interventions is that they are less likely to be able to reach some of the most vulnerable groups who do not have a regular address, such as those who are homeless.

### Comparison with existing literature

The intervention is only a partial solution to the problem of incongruous consultation behaviour in primary care particularly bearing in mind the pressures on capacity. Other studies have explored the feasibility and acceptability of targeted in-person health checks for improving awareness of cancer symptoms,^30^^,^^31^ and will explore impact on symptom awareness.^32^

The targeted promotion of cancer awareness and help seeking for specific symptoms may help increase and modify help-seeking behaviours, and reduce inequity in both access to care and cancer survival in a way that mass campaigns may not.^9^

### Implications for practice

Similar interventions may have applications in mitigating other health inequalities by facilitating more equitable access to other areas of primary care (such as pharmacies and psychological wellbeing services).^33^^,^^34^ Other applications may include: facilitating more equitable access to support for health and wellbeing promotion; for the primary, secondary, and tertiary prevention of disease, such as recent letters advising those at increased risk of COVID-19 to shield themselves by staying at home;^35^ and signposting recipients to the Every Mind Matters website.^36^

During the COVID-19 pandemic, there have been fewer referrals to secondary care and fewer diagnoses of cancer. Patients are balancing the risks of infection with the risks of delayed diagnosis. This need may, in part, be addressed through initiatives as described here, though such an intervention will have to be timed to match re-opening of diagnostic services.

This trial demonstrates the possible potential of an inexpensive, targeted, postal symptom-awareness intervention for altering consultation behaviour and reducing barriers to help seeking in general practice.

There is a need to evolve similar interventions with the potential to support a wider range of patients (such as those who may not be registered with a GP), and to explore whether there is a dose response to a series of communications or any impact on diagnoses. This is particularly relevant in the UK given the commitment of the NHS to providing equitable care.^37^
